# Emerging Roles of FTO in Neuropsychiatric Disorders

**DOI:** 10.1155/2022/2677312

**Published:** 2022-04-26

**Authors:** Rui Chang, Zeyi Huang, Size Zhao, Ju Zou, Yukun Li, Sijie Tan

**Affiliations:** Department of Histology and Embryology, Institute of Clinical Anatomy & Reproductive Medicine, School of Basic Medicine, Hengyang Medical School, University of South China, 421001 Hunan, China

## Abstract

FTO (fat mass and obesity associated) is a recently discovered gene related to obesity and expressed in various tissues of the human body, especially with high expression in the brain. Earlier studies have found that FTO is involved in several biological processes, including brain development and function. In particular, recent studies have found that FTO is a demethylase of N6-methyladenosine (m6A) and it can affect neurological function through the m6A modification of mRNA. At present, a number of studies have shown that FTO is associated with many neuropsychiatric disorders. This paper reviews the discovery, structure, function, and tissue expression of FTO followed by discussing the relationship between FTO and neuropsychiatric diseases. In addition, the potential roles of FTO gene in drug addiction, major depression (MDD), and schizophrenia (SCZ) through regulating m6A modification of dopamine related genes were also highlighted.

## 1. Introduction

The prevalence of neuropsychiatric diseases has been continuously increasing in the past few decades. For this reason, these diseases have become a major public health problem worldwide [1]. At present, studies have shown that neuropsychiatric diseases such as drug addiction, major depression (MDD), and schizophrenia (SCZ) have become a global problem. Hence, novel targets for these neuropsychiatric diseases are needed.

Among these neuropsychiatric diseases, MDD is a universal, chronic, disabled, and multidimensional mental illness. Although the prevalence of MDD has been increasing, its pathogenesis and etiology are still unclear. Moreover, the research on antidepressant treatment for MDD patients, in particular, at the molecular level is difficult, as it is not easy to model MDD at cellular and/or molecular level. Some antidepressant drugs, such as vortioxetine, have been shown to directly and independently alleviate the symptom of MDD temporally, but the recurrence rate is high. Therefore, further research is needed to find more effective treatments for MDD [2].

The pathophysiological research of SCZ mainly focuses on dopaminergic and glutamatergic neurotransmission disorders, but the clinical treatment is limited [3–5]. Although the current antipsychotic drugs have greatly reduced the incidence rate of SCZ, they have poor efficacy and serious side effects [6].

Addiction is a chronic and recurrent disease that requires long-term treatment. Studies have found that there are two main neural pathways related to addiction: (1) the midbrain dopamine reward pathway, which is crucial for survival, will change physiologically due to drug abuse, leading to uncontrolled drug craving. (2) The prefrontal cortex is a decision to control inappropriate reward responses, which can also be changed by drug abuse. At present, relapse is the main challenge for clinicians who treat patients with addictive diseases [7].

N6-methyladenosine (m6A) is the most abundant form of methylation modification in eukaryotic mRNA, and it is also the most studied RNA modification method. In mammals, m6A is installed by the methyltransferase complex, including methyltransferase-like3 (METTL3), methyltransferase-like 14 (METTL14), Wilms tumor 1-associated protein (WTAP), KIAA1429, RBM15 (RNA-binding motif protein 15) [8], and its paralog (RBM15B), whereas it can be removed by the demethylases fat mass and obesity associated (FTO) and alkylation repair homolog protein 5 (ALKBH5) [9]. Studies have shown that m6A-related regulatory proteins, in particular the FTO, perform important roles in the synaptic transmission, axon regeneration, neural stem cell self-renewal, and cerebral cortex development [10–14]. In addition, epigenetic studies have shown that m6A/mRNA modification not only plays an important role in the accumulation of learning and memory [15] but also crucial for regulating stress-induced behaviors [16, 17]. Moreover, Koranda et al. [17] found that the downregulation of m6A methylation in substantia nigra impairs striatal-mediated learning and alters dopamine signaling.

FTO is the first obesity susceptibility gene identified by a genome-wide association study and showed that multiple single nucleotide polymorphisms (SNPs) of FTO gene were associated with obesity risk [18, 19]. Studies in mouse models have shown that FTO played a key role in regulating fat mass, fat formation, and body weight [20–22]. Further studies have found that FTO played a key role in the metabolism of many tissues, including brain, brown adipose tissue (BAT), white adipose tissue [23], and liver [24].

Although multifactor and multiple genes have been involved in the pathogenesis of common psychiatric disorders such as addiction [25], depression [26–28], and schizophrenia [29] [30] [31], the etiology of these diseases has not been fully understood. FTO is a recently discovered gene associated with obesity [32, 33], but there is already evidence that FTO plays a role in these psychiatric disorders. For example, a recent study reported that FTO in the medial prefrontal cortex was related to fear memory [16, 34], while another study showed that FTO controls the dopaminergic circuit in the midbrain, which is the key pathway for learning, rewarding, motor function, and eating behavior [35–38]. FTO gene variations were also related to MDD and depressive symptoms. Rivera et al. [39] investigated the association between FTO and BMI within the context of MDD and found that subjects with a history of depression changed the effect of FTO on body mass index (BMI), which indicates that FTO is involved in the mechanism of association between emotional disorders and obesity. In addition, Zhang et al. [40] found that weight loss may be accompanied by a decrease in neuronal activity related to anxiety, which in turn reduces anxiety and reduces the occurrence of depression. Similarly, King et al. [41] also confirmed through experiments that the severity of depression after bariatric surgery for severe obesity is also significantly reduced. The above results indicate that FTO can regulate the dopamine circuit related to learning, memory, and reward in the brain. On the other hand, FTO, as an obesity-related gene, can also inhibit the occurrence of depression through reducing the BMI.

This article reviews the role of FTO in the development of addiction, depression, and SCZ and discusses the mechanism by which FTO may influence the occurrence and development of these neurological diseases.

## 2. Fat Mass and Obesity Associated (FTO)

### 2.1. Discovery of FTO Gene

FTO was firstly found in 2007 when Frayling et al. conducted a genome-wide association in the type 2 diabetes (T2DM) subjects in Europe and found that the SNP in the first intron of the FTO gene was associated with BMI, indicating that FTO was closely related to obesity [42]. FTO is also the first obesity susceptibility gene identified in the genome-wide association studies. Therefore, the gene was officially named fat mass and obesity-related protein (FTO).

### 2.2. Location and Distribution of FTO Genes

Genomics studies have shown that FTO genes only exist in the genomes of vertebrates and a few marine algae genomes, but not in the bacteria, fungi, protozoa, invertebrates, and most plants [43, 44]. The human FTO gene is located on the long arm of chromosome 16, with a total length of about 400 kb, including 9 exons and 8 introns. Frayling et al. [42] used real-time fluorescent quantitative PCR to detect the expression of FTO gene in fetal and adult tissues and found that FTO is widely expressed in fetal and adult tissues, with the highest expression in the brain. Madsen et al. [45] investigated the expression of FTO gene in various tissues of pigs, and the results showed that the expression level of FTO gene in the brain was 7-12 times higher than that in peripheral tissues, and the expression level of FTO gene in cerebellum was significantly higher than that in the hippocampus and cerebral cortex. Fredriksson et al. [46] showed that the FTO gene is mainly expressed in neurons, but not in astrocytes and glial cells.

### 2.3. The Function of FTO Gene

Since it was discovered as an obesity-related gene, most of the existing studies have focused on the roles of FTO in obesity [32, 33]. However, increasing studies have found the abundant expression of FTO protein in the brain, especially in neurons [47–49]. In addition, it was shown that FTO can regulate the activity of dopaminergic circuits of the midbrain. Inactivation of the FTO gene weakens neuronal activity and behavioral responses that depend on dopamine receptor type 2 (D2R) and type 3 (D3R) (collectively called D2-like receptors) [50]. FTO also regulates defects in dopaminergic neurotransmission caused by arsenate [51]. Walters et al. [52] found that FTO plays an important role in the formation of hippocampal-dependent memory in mice. The decrease in FTO protein observed shortly after the situational fear reflex indicates that FTO may inhibit memory formation. Through in-depth research, the researchers found that when the FTO gene in the mouse prefrontal cortex was knocked out, the m6A intensity of several fear-related genes in neurons was significantly increased, and the consolidation of fear memories was enhanced [16]. In addition, recent studies have found that FTO is an RNA N6-methyladenosine (m6A) demethylase. As an important regulator of m6A-marked transcripts, FTO is very important for mRNA alternative splicing and gene expression [53]. Considering the regulatory role of FTO in learning and memory [54], we speculate that FTO might play an important role in normal cognitive function and psychiatric diseases by regulating dopaminergic function through mRNA methylation modification.

## 3. FTO in Addiction

### 3.1. Definition and Harms of Drug Addiction

Drug addiction (addiction), also known as drug dependence, is a neuropsychiatric disorder characterized by repeated drug taking regardless of harmful consequences [55]. Psychoactive substance addiction has become an important factor that restricts economic development, threatens social security, and endangers public health. Nicotine and alcohol are the most common abused addictive substance. Nicotine can activate neuronal nicotinic acetylcholine receptors (nAChRs) in the brain's natural reward system (midbrain limbic pathway). This pathway is composed of dopaminergic neurons originating from the ventral tegmental area (VTA), which project to the nucleus accumbens (NAc), prefrontal cortex (PFC), amygdala, and hippocampus [56, 57].When nAChRs is activated, it will increase dopaminergic firing and release and project dopamine (DA) neurons to various tegmental areas, especially NAc, an area that can produce rewarding and strengthening effects [56, 58]. In addition, nicotine can also regulate the release of gamma-aminobutyric acid (GABAergic) interneurons and reduce the inhibition of DA neurons [59], causing the DA level in NAc area to increase, producing reward effects (such as euphoria, etc.), and ultimately leading to the body's psychological dependence on nicotine.

Alcohol affects several kind of neurotransmission systems in the brain [25]. Generally speaking, acute alcohol will enhance inhibitory transmission, upregulate the GABAergic system, and impair glutamatergic function, thereby disturbing the balance between excitatory and inhibitory synaptic input. In order to restore this balance, the brain will form long-term drinking and eventually become addicted, causing it to undergo neuroadaptive changes, resulting in a decrease in *γ*-aminobutyric acid (GABA) energy and an increase in glutamate (Glu) energy activity. At the same time, the functions of other neurotransmitters are also changed by the presence of alcohol, including glycine, adenosine, serotonin, and dopamine. In addition, alcohol also has a significant effect on the endogenous opioid system, nicotinic cholinergic transmission, and the endocannabinoid system.

### 3.2. The Relationship between FTO and Addiction

In recent years, studies [60] have found that BMI/obesity susceptibility genes are abundantly expressed in the brain, especially in the insular and substantia nigra, two brain regions related to addiction and rewarding [61]. First of all, agranular insula of the insular contains a high density of dopamine D1 receptors and receives dopaminergic innervation, and there has been evidence that its dopaminergic effect may also be involved in addiction [62]. Secondly, the substantia nigra, located in the basal ganglia of the midbrain, is also involved in addiction, motivation, and reward-seeking behavior and exhibits a high density of dopaminergic neurons [63, 64]. Researchers also found that FTO is the gene most related to BMI in these two brain regions [65]. FTO gene is also one of the most associated with obesity which has been identified as a risk gene for addiction [65]. In a behavioral and functional magnetic resonance imaging study, researchers demonstrated that FTO deficiency specifically impairs the control of neuronal activation mediated by the dopamine receptor D2/3R [66]. It was also found that FTO deficiency resulted in increased m6A modification of specific mRNA of D2/3R signaling key components (including D3R and GIRK2 channels), which reduced their translation and affected dopamine dependent regulation of reward sensitivity [50], suggesting that FTO may affect the function of dopamine in addiction by reducing the m6A modification in related genes. In addition, the dynamic causal model also confirmed that the FTO gene mutation regulated the connectivity of the basic reward circuit in the middle striated prefrontal region, which indicated that genetic susceptibility changed reward processing not only in obesity but also in other D2R-dependent impulse control diseases, such as addiction [66].

In addition, many studies have reported that FTO participates in the occurrence of addiction through modulating the dopaminergic circuits. In 2007, it was reported that behavioral changes related to human FTO mutations were associated with altered in dopaminergic transmission [61], and then some authors found that FTO SNP mutations rs9939609 may be negatively related to alcohol consumption [65]. In 2011, studies reported that FTO controls the dopaminergic circuit in the brain [36], which has been shown to be associated with drug and alcohol abuse, as well as with the urge to overeat in people with higher levels of BMI and obesity [67]. Specifically, FTO may affect the occurrence of addiction through the dopaminergic circuit. In 2013, a study reported that both addiction and obesity increase dopamine in the midbrain reward system [68]. However, studies have shown that abnormalities in this reward system are likely to be caused by complex interactions of genetic and environmental effects [69]. These findings suggest that FTO may be involved in the reward and enhancement of drug addiction through the dopaminergic circuit, suggesting that m6A may play an important role in it.

## 4. FTO in Depression

### 4.1. Definition and Harm of Depression

Depression is a mental disease that seriously endangers human's physical and mental health. It is a common emotional disorder syndrome. It is a kind of mood disorder characterized by significant and persistent depression, mental retardation, and cognitive impairments. In 2010, depression was found to be the second leading cause of disability for many years in the global burden of disease study. By 2030, depression will rise to the top cause of global disability [70]. Depression not only damages the health of patients and reduces the quality of life but also brings a huge burden to the family and society [71].

### 4.2. The Mechanism of Depression

The pathogenesis of depression is very complex and has not been fully understood. In exploring the pathogenesis of depression, the authors have put forward some hypotheses including monoamine hypothesis of depression [26–28], neuroendocrine hypothesis [72], and hippocampal neuronal aplasia hypothesis [73]. Among them, the monoamine hypothesis is one of the most accepted hypotheses in MDD [26–28]. It has been hypothesized that decreased concentration or function of monoamine neurotransmitters, including dopamine (DA), norepinephrine (NE), and serotonin (5-HT), in the synaptic gap of the central nervous system is the biological basis of depression. In addition, there is a link between depression and obesity, but this link has been controversial: many studies have shown that overweight and obesity are associated with adverse mental health outcomes, although other studies have shown that higher BMI indicates a lower risk of depression [74]. In recent years, more and more evidence show that the abnormality of immune-inflammatory pathway is involved in the pathophysiological process of MDD [75]. Through a cross-sectional study, Passos et al. found that some MDD patients showed changes in the peripheral immune system, such as impaired cellular immune function and increased levels of proinflammatory cytokines [76]. Through further research, Han et al. found that patients treated with cytokines tend to have depressive symptoms [77].

### 4.3. The Relationship between FTO and Depression

In 1975, Andersen et al. firstly proposed that DA may be involved in the occurrence of depression and found that the function of DA was decreased in the brain of depression subjects [26]. Subsequently, Han et al. found that the presynaptic membrane transporter dopamine transporter (DAT) of dopaminergic neurons can reuptake the DA from the synaptic terminals to maintain the physiological concentration of DA in the synaptic gap and found that the density of DAT in the striatum of patients with depression is significantly higher than that of normal controls [77]. Excessive DAT can increase the reuptake of terminal DA and reduce the level of DA in the synaptic gap, which leads to depression [77]. Recent studies in rodent models have shown that the conditioned loss of FTO in dopamine neurons specifically impairs neuronal activation through dopamine receptors 2 and 3 (Drd2 and Drd3) [78]. The findings of this study suggest that FTO may lead to depression by downregulating the level of dopamine. However, whether this downregulation is attributed to the demethylase function of FTO merits further investigations. Previously, it has been reported that higher body mass index (BMI) will increase the risk of depression. Some authors have proposed that FTO may participate in the occurrence of depression by increasing BMI as FTO is an obesity susceptibility gene [79].

In recent years, Samaan et al. [80] conducted a more in-depth study of FTO and found that the FTO rs9939609A variant is associated with a higher BMI, but with a protective effect on depression. In another study, Yao et al. showed that the FTO single nucleotide polymorphism rs9939609 is not associated MDD using meta-analysis in Asian populations [81]. In addition, it has also been reported that FTO can affect intestinal microflora, and the absence of FTO produces an iconic intestinal microflora, which may reduce the content of lipopolysaccharide (LPS) in serum, thereby inhibiting the activation of proinflammatory cytokines induced by stress. Importantly, FTO^+/-^ mice are less sensitive to stress stimulation, and FTO knockout mice show decreased anxiety and depressive behaviors [82], suggesting that deletion of FTO may affect brain functions by affecting the intestinal flora and then lead to the occurrence of depression.

## 5. FTO in Schizophrenia

### 5.1. Definition and Harm of Schizophrenia

Schizophrenia is a chronic mental disability disease. The symptoms of STZ can be roughly divided into three categories, namely, positive symptoms, negative symptoms, and cognitive impairment [83]. Positive symptoms refer to exaggeration or distortion of normal functional behavior, mainly including thinking disorders, delusions, hallucinations, and repeated bizarre actions; negative symptoms refer to the weakening or loss of normal functional behavior, such as reduced euphoria experience, lack of thinking and speech, and emotional apathy; cognitive impairment includes difficulty in concentration, poor execution, and working memory disorders. The prevalence of schizophrenia was around 1% in the general population [84]. In the schizophrenia subjects, the incidence of metabolic syndrome was 2-3 times higher than that of unaffected subjects [85]. In addition, cardiovascular disease was the main reason for the high mortality rate of schizophrenia [86]. It can have a devastating impact on patients and caregivers and bring huge costs to the health-care system [87].

### 5.2. The Mechanism of Schizophrenia

At present, although the pathogenesis of schizophrenia is not clear, the dopamine hypothesis [29], *γ*-aminobutyric acid hypothesis [30], and glutamate hypothesis [31] have been proposed. In recent years, the dopamine hypothesis is more popular for the pathogenesis of schizophrenia because the increased activity of the dopamine system in the inner cortex is related to the positive symptoms of schizophrenia. Imaging studies have shown that amphetamine stimulants can significantly increase the release of dopamine in the striatum of schizophrenic patients [88], and increasing the release of dopamine can also aggravate the positive symptoms of schizophrenia [89], which also provides more theoretical support for the dopamine receptor hypothesis. Abnormal metabolism function represents another risk factor for schizophrenia. For example, Chen et al. evaluated the cognitive function and clinical symptoms of 158 schizophrenic patients by using MCCB and BPRS and found that the neurocognitive function of schizophrenic patients may be related to the deficiency of metabolic state [90]. Kassm et al. found that almost half of the schizophrenic patients had metabolic syndrome in a cohort study of schizophrenic patients aged 55 or over (nasty 353) [91], which further supported that metabolic syndrome was involved in the occurrence of schizophrenia. However, Tay and Lee analyzed anthropometric parameters and fasting blood samples for metabolic measurements in 81 patients with schizophrenia and found no significant association between schizophrenia and metabolic syndrome [92].

### 5.3. The Relationship between FTO and Schizophrenia

It is suggested that metabolic syndrome plays an important role in schizophrenia. In the schizophrenia patients, the incidence of metabolic syndrome (MetS), a group of biochemical and physiological risk factors for cardiovascular disease (CVD), was significantly higher than that of the general population [93, 94]. Patients who receive long-term antipsychotic (AP) drug often gain weight, especially some atypical AP [95, 96], and may lead to obesity, impaired glucose tolerance, diabetes, and CVD [97]. The typical AP mainly targets the dopamine receptor, while the nontypical AP mainly targets the serotonin receptor [98], indicating that the abnormality of monoamine neurotransmitters may lead to metabolic syndrome and then affect schizophrenia.

Moreover, a large number of studies have shown that the gene polymorphism of FTO is closely related to metabolism disorders such as obesity [99, 100] and T2D [101, 102]. Considering the close relationship between MetS and schizophrenia, Malan-Muller et al. [103] proposed that the polymorphisms of FTO gene may be involved in the occurrence of metabolic syndrome and lead to schizophrenia. In 2014, a study [104] reported that there was a significant correlation between FTO rs9939609 and MetS in chronic schizophrenic patients treated with AP and that the SNP rs9939609 of FTO gene was related to the occurrence of MetS in patients receiving APs. Finally, it is concluded that FTO genotype has a long-term effect on MetS or obesity susceptible patients [104]. In the same year, it was found that SNPs rs9939609 and rs8050136 of FTO gene may play an important role in weight gain after 6 months of risperidone treatment [105]. In 2011, Tiwari et al. [106] found that the SNP rs9922047 polymorphism of FTO gene was associated with the percentage of weight gain in patients with schizophrenia. In 2010, a study [107] reported that there was a similar weight gain among the three genotypes of FTO gene SNP rs9939609 in schizophrenic patients after one year of antipsychotic treatment. From the above results, we found that the polymorphism of FTO gene may lead to obesity and then lead to metabolic syndrome and participate in the occurrence of schizophrenia. In addition, Tiwari et al. genotyped four single nucleotide polymorphisms (rs9939609, rs8050136, rs1421085, and rs9930506) of first-episode schizophrenia by polymerase chain reaction-restriction fragment length polymorphism (PCRRFLP) and direct sequencing [105]. It was found that FTO gene polymorphism, especially rs9939609, may be associated with weight gain after risperidone treatment in Han Chinese patients with first-episode schizophrenia. Although some scholars have found that there is no correlation between FTO genotype and weight gain in schizophrenic patients who initially did not take drugs [108], the above-mentioned studies suggest that FTO gene polymorphism may be involved in the occurrence of schizophrenia by affecting body weight, through these findings.

## 6. Authors' Insight on the Topic

FTO gene is a gene related to fat metabolism and obesity. It is widely distributed in various tissues and highly expressed in brain structures, especially in the hypothalamus, which controls appetite. Studies have shown that FTO is a demethylase that regulates the level of m6A in mRNA and might play key roles in the occurrence and development of addiction, depression, schizophrenia, and other diseases. The relationship between FTO and activity of dopamine system is noteworthy because it can affect the outcome of a variety of neuropsychiatric diseases. The increased activity of dopamine system is closely related to the development of addiction and schizophrenia. On the contrary, the decrease in the activity of dopamine system may aggravate the occurrence of depression. The dopamine system is the main rewarding system in the brain and consists of several key molecules such as DAT, DRD1-3. Although previous study has shown that FTO can directly affects the activity of DRD2 through removal of m6A, whether FTO could modulate other proteins in the dopamine system is not clear. Further studies exploring the functional roles of FTO in the dopaminergic system could be helpful for better understanding the development of these neuropsychiatric diseases and provide novel targets for them.

## Abbreviations

FTO: Fat mass and obesity associated
m6A: N6-methyladenosine
MDD: Major depression
SCZ: Schizophrenia
RRACH: R: purine; A: m6A; H: nonguanine
ALKBH5: Alkylation repair homolog protein 5
SNPs: Single nucleotide polymorphisms SNPs
BAT: Brown adipose tissue
NMDA: N- methyl aspartate
ACh: Acetylcholine
T2DM: Type 2 diabetes
GABAergic: Gamma-aminobutyric acid
MetS: Metabolic syndrome
AP: Antipsychotic
BMI: Body mass index
ANSCs: Adult neural stem cells
mPFC: Medial prefrontal cortex
nAChRs: Nicotinic acetylcholine receptors
VTA: Ventral tegmental area
NAc: Nucleus accumbens
PFC: Prefrontal cortex
DA: Dopamine
NE: Norepinephrine
DAT: Dopamine transporter
DRD2: Dopamine receptor 2
DRD3: Dopamine receptor3
LPS: Lipopolysaccharide
CVD: Cardiovascular disease
PCRRFLP: Polymerase chain reaction-restriction fragment length polymorphism.
